# An adaptive term proximity based rocchio’s model for clinical decision support retrieval

**DOI:** 10.1186/s12911-019-0986-6

**Published:** 2019-12-12

**Authors:** Min Pan, Yue Zhang, Qiang Zhu, Bo Sun, Tingting He, Xingpeng Jiang

**Affiliations:** 1 National Engineering Research Center for E-Learning, Central China Normal University, Wuhan, 430079 China; 2 School of Computer and Information Engineering, Hubei Normal University, Huangshi, Hubei Normal University, Huangshi, 435002 China; 3 School of Computer, Central China Normal University, Wuhan, 430079 China

**Keywords:** Clinical retrieval, Term proximity, Query expansion, Pseudo relevance feedback

## Abstract

**Background:**

In order to better help doctors make decision in the clinical setting, research is necessary to connect electronic health record (EHR) with the biomedical literature. Pseudo Relevance Feedback (PRF) is a kind of classical query modification technique that has shown to be effective in many retrieval models and thus suitable for handling terse language and clinical jargons in EHR. Previous work has introduced a set of constraints (axioms) of traditional PRF model. However, in the feedback document, the importance degree of candidate term and the co-occurrence relationship between a candidate term and a query term. Most methods do not consider both of these factors. Intuitively, terms that have higher co-occurrence degree with a query term are more likely to be related to the query topic.

**Methods:**

In this paper, we incorporate original HAL model into the Rocchio’s model, and propose a new concept of term proximity feedback weight. A HAL-based Rocchio’s model in the query expansion, called HRoc, is proposed. Meanwhile, we design three normalization methods to better incorporate proximity information to query expansion. Finally, we introduce an adaptive parameter to replace the length of sliding window of HAL model, and it can select window size according to document length.

**Results:**

Based on 2016 TREC Clinical Support medicine dataset, experimental results demonstrate that the proposed HRoc and HRoc_AP models superior to other advanced models, such as PRoc2 and TF-PRF methods on various evaluation metrics. Among them, compared with the Proc2 and TF-PRF models, the MAP of our model is increased by 8.5*%* and 12.24*%* respectively, while the F1 score of our model is increased by 7.86*%* and 9.88*%* respectively.

**Conclusions:**

The proposed HRoc model can effectively enhance the precision and the recall rate of Information Retrieval and gets a more precise result than other models. Furthermore, after introducing self-adaptive parameter, the advanced HRoc_AP model uses less hyper-parameters than other models while enjoys an equivalent performance, which greatly improves the efficiency and applicability of the model and thus helps clinicians to retrieve clinical support document effectively.

## Background

### Introduction and motivation

Retrieving more relevant articles for the clinician is helpful to improve their decision-making on the diagnosis, treatment and test of patients [1].The TREC Clinical Decision Support (CDS) tracks provides corpora for such systems and encourages the information retrieval tools and resources needed to implement these systems. The real hospital notes contain a lot of abbreviations and other language styles^1^, and extracted by clinicians as queries. A note is usually longer than a summary or a description. All of those will bring new challenges for traditional retrieval systems. Thus, a clinician rewrites his or her notes in a standard report unreasonably. Instead, to reduce the time burden of clinician, the task of information retrieval system should be to operate notes directly. We retrieve full-text biomedical articles for answering questions related to one of three generic clinical information needs: Diagnosis, Test and Treatment.

To address the challenge, we use Pseudo Relevance Feedback (PRF) technique. PRF is a very famous query extension technology [2–6]. It assumes that the first retrieval top-ranked documents are relevant to the query. Then PRF refines the potentially related terms weight and adds to the original queries. Although the traditional PRF has been proved to be very effective [7, 8], it still fail in some classic IR tasks. The expansion terms are selected according to the candidate term frequency or the term distributions of the feedback documents are irrelevant.

Integrating term proximity information into PRF can improve retrieval efficiency and become a hot spot of research [9]. The Hyperspace Analogue to Language (HAL) [10] is a mental theory of term meaning calculation model, only considering the context of words, immediately surrounded by a given word. Most of these researches focus on the term proximity in the original query and apply it to the sorting documents [11–18]. However, most traditional term proximity methods ignore the significance of term frequency.

The main contributions of this paper are as follows:

Adapting a new concept of proximity information weight, we propose a proximity based PRF model, called HRoc.

Introducing three normalization methods to make a fair comparison.

Adapting the adaptive function to make the length of sliding window (denote by D value) of HAL model dynamically adjust according to the length of the document and increasing the universality of the model.

Our proposed model has been proved to be effective by TREC clinical medicine collections.

### Related work

The CDS track complements the previous TREC tasks inspired by biomedicine [1], specifically, the genomics and medical records tracks. The CDS track has been heavily inspired by the TREC genomics [19], medical records [20] tracks and the medical case-based retrieval track of Image-CLEF [21]. They all shown great interest in medical ad-hoc retrieval. There are no reusable, uncertified set of medical records, in [22, 23], short case reports, proposed that the real medical records should be represented by idealized method. For a given case report, follow-up participants retrieve full-text biomedical articles and answer questions related to several types of clinical information needs. The 2016 CDS focuses on topic query expansion modeling by actual patients note [24–26].

In Information Retrieval (IR) process, original queries may lead to the absence of some important terms information. PRF method is a common but effective technique for achieving better retrieval performance in [2, 7, 8, 27], which the semantic relationship between the added terms and the original query terms is considered, including these defined relations in Rocchio’s model. It brings better result. Then, many other relevance feedback techniques and algorithms were proposed and most of them were derived under the Rocchio’s framework. For example, A famous and successful automatic PRF algorithm is proposed in okapi system by Robertson *et al* [28]. A feedback framework based on proximity (called proc) is proposed by Miao et al, which includes different proximity measures to estimate the correlation and importance of candidate options [29]. Ye and Huang propose a unified model (TF-PRF) to capture local saliency of related candidate in feedback documents [30]. These two models are strong baselines, and used for comparison in our experiments. In addition, many other competitive approaches have obtained significant performance in improving retrieval effectiveness [4*,*^31^]. Since they are not that related to our research methods, we do not introduce them in detail.

Recently, plenty of work has been studied to integrate term proximity and other relationships into existing IR models. In [32], the authors introduced a pseudo term to the model in the Dirichlet language model, the approximate centrality of query terms is used as a parameter. LV and Zhao integrated location and proximity information into the language model from a second perspective [33]. These relations play an important role in IR field. Mbarek *et al* have obtained significant performance in improving retrieval effectiveness [34]. Rasolofo *et al* use proximity measurement in combination with the Okapi probabilistic model [17]. Peng *et al* incorporate term dependency in the DFR framework [35]. Metzler *et al* developed a general and formal framework for modeling term dependency through Markov random fields, and developed a new method to train the model, which directly maximizes the average accuracy rather than the availability of training data. [36]. Zhao *et al* use Triangle Kernel functions in information retrieval applications [37]. In this paper, we propose three HAL-based co-occurrence PRF models, in which we integrate the approximate weight information of a term into the traditional PRF model: Rocchio model. In our method, we estimate their weights by considering the distance between the candidate expansion and the query item. In addition, we introduce three normalization methods for a new concept of proximity-based term weighting and an adaptive function to make the D value dynamically adjust according to the length of the document.

## Methods

### Traditional pRF models

Our study based on a classic traditional PRF model. In this section, we will first briefly revisit the Rocchio’s model. The Rocchio’s model is a classical framework to realize pseudo relevance feedback representation, which incorporates the information of pseudo relevance feedback in the first-pass retrieval [27]. In PRF, the feedback documents often contain relevant and irrelevant documents, but the irrelevant in the Rocchio equation is ignored. Finally, the query is realized by the linear combination of the initial query vector and the feedback document vector.

1 $$ {Q_{1}} = a * {Q_{0}} + \beta * {\Sigma_{r \in R}}\frac{r}{{\left| R \right|}}  $$

where *Q*_0_ and *Q*_1_ represent the original and new query vectors respectively, |*R*| represents the number of the feedback documents, *r* is the expansion terms weight vector for feedback documents, *α* and *β* represent the original query weight and the related documents weight respectively. In Eq. (1), we can notice that *α* and *β* are actually constant values, which control how much we rely on the original query and the feedback information. In practice, we can always set *α* at 1.0, and only study *β* until we get better performance.

However, traditional Rocchio’s model does not capture the relationship between an original query term and a candidate term. Generally, a candidate term is supposed to be relevant to the query topic if it occurs near to a query term.

### HAL method for term co-occurrence weight

**Fig. 1:**
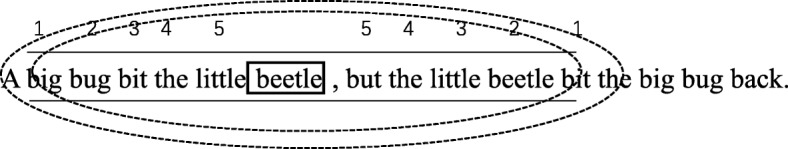
An example for counting local co-occurrences

The basic motivation is that when a person encounters a new concept, he or she is inclined to infer its meaning from other concepts occurring within the same context. For example, a document that contains both “heart disease” and “China” is irrelevant to the topic “heart disease in China” when these two terms are not close to each other in the context. Therefore, term proximity is effective to discriminate against these types of documents. Vechtomova et al. using multiple distance factors and mutual information to select query extension from Windows Environment [38]. HAL model constructs a high-dimensional vector for each word by simply treating the context of a given query word as a close word [10,39,40]. HAL method begins by producing a co-occurrence matrix |*V*|∗|*V*| for each term in a specified vocabulary |*V*|. This process of counting local co-occurrences is illustrated in Fig. 1.

**Fig. 2:**
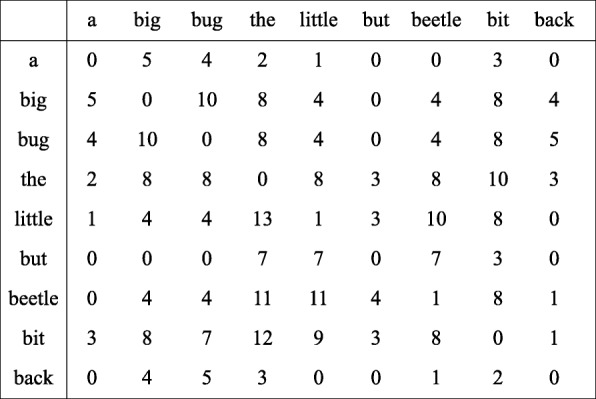
The co-occurrence matrix contain after HAL-style weighting of the counts from the sliding window

It is assumed that the length of a document is 18, and the *D* value is set to 5. For each word *a*, a proximity relation can be generated between *a* and every word *b* which occurs close to *a*. Then their distance strength can be calculated as follows. If *B* occurs adjacent to *A*, the strength is 5. Then if *B* and *A* are separated by a word, it would get the strength of 4, and it also drop to the intensity of 1. The element *W*_*a*,*b*_ (row *A*, column *B*) of the symbiotic matrix contains the weighted sum of all occurrences of *B* close to *A*. The co-occurrence matrix contains after HAL-style weighting of the counts from the sliding window in Fig. 2.

The weighting of each co-occurred term is accumulated over the whole corpus. We adapt the original HAL model similar as in [39,40]. Then, the weight calculation of each term can be represented by a semantic vector within a specified distance.

2 $$ {HAL(t,q)} = {\sum\nolimits}_{l=1}^{\left| D \right|}{w(l)*p(t,l,q)}  $$

where *l* is the distance from query term *q* to term *l*, *p*(*t*,*l*,*q*) is the co-occurrence frequency within the sliding windows when the distance equals *l*, then *w*(*l*)=*D*−*l*+1 denotes the strength. In this paper, we need to construct vector for term in document, which denotes a proximity relationship with the entire query. Intergrading the average proximity information into the query term weight could make a better result. Then we also take into account the distinction factor of different query terms. Therefore, each dimension is the specific representation vector weight of the query term, which can be calculated by the inverse document frequency formula below, and then the proximity-based term weight *W*_*HAL*_(*t*,*q*) in the method is computed as follows:

3 $$ {W_{HAL}(t,q)} = {\sum\nolimits}_{i=1}^{\left|Q_{0}\right|}{{HAL(t,Q_{0})}*IDF(q_{i})}  $$

The weighted HAL model combines the two elements of term distance and co-occurrence frequency at the same time. It is the first time that the HAL model was adopted to measure the proximity between the query terms and the candidate terms in such way, and then used in the field of medical retrieval.

### A hAL-based pRF model

We take into account the significance of TF-IDF and proximity information, hence propose a HAL-based co-occurrence model for PRF via integrating a term’s co-occurrence information into traditional PRF models for expansion terms selection. Formally, let *Q*_0_={*q*_1_,*q*_2_,…,*q*_*m*_} represents the original query given by the user, a new query *Q*^′^ will be generated by a method HRoc as follows:

4 $$ {Q'}=(1-\alpha)*Q_{0}+\alpha*\left((1-\beta)*\Sigma_{r \in R}\frac{r}{{\left| R \right|}}+\beta*\Sigma_{r' \in R}\frac{r'}{{\left| R \right|}}\right)  $$

where *α* and *β* are tuning coefficients of 0 to 1.0. *α* is constant value for tuning the contribution weight between the original query and the feedback information, and *β* is constant value for balancing the contribution weight between the feedback information measured through common term frequency or term distributions and the feedback information measured through the corresponding term co-occurrence.

In Eq. (4), *Q*^′^, *Q*_0_ and |*R*| have the same meanings with those in Eq. (1). Parameter *r* is the vector of expansion term weight computed with BM25, and *r*^′^ is the vector of expansion term proximity co-occurrence weight for feedback documents, which reveals the relationship between a candidate term and a query topic.

5 $$ {}\begin{aligned} {Q'}=(1-\alpha)*Q_{0}+\alpha*\left((1-\beta)*\Sigma_{r \in R}\frac{r}{{\left| R \right|}}+\beta*\Sigma_{r' \in R}\frac{{W_{HAL}(t,q_{i})}}{{\left| R \right|}}\right) \end{aligned}  $$

In order to better compare with advanced model, we design three normalization methods of term proximity weight in next part.

### Normalization methods

Normalization is convenient for data processing. We get weight score ranking in Eq. (5). Due to the big difference between the multiplicative values, the effect is certainly not good if it is directly integrated into the Rocchio’s model. To solve this problem, we adopt three normalization methods to optimize the weight score. The formula representation after the introduction of normalization is shown in Eq. (6):

6 $$\begin{array}{*{20}l} {Q'}=(1-\alpha)*Q_{0}&+\alpha*\left((1-\beta)*Norm\left(\Sigma_{r \in R}\frac{r}{{\left| R \right|}}\right)\right.\\ &+\left.\beta*Norm\left(\Sigma_{r' \in R}\frac{{W_{HAL}(t,q_{i})}}{{\left| R \right|}}\right)\right) \end{array} $$

Three normalization methods are presented in Table 1.

**Table 1:** The three kinds of normalization method

*Norm*(*t*)		
*norm*_1_(*t*)	*norm*_2_(*t*)	*norm*_3_(*t*)
*t*−*min*(*t*)/*max*(*t*)−*min*(*t*)	${t}/{\sqrt {sum(t^{2})}}$	*t*/*max*(*t*)

We take methods of *norm*_1_(*t*), *norm*_2_(*t*) and *norm*_3_(*t*) to process data respectively and *t* represents the different weight values in Eq. (6). We call them HRoc1, HRoc2 and HRoc3 by using three normalization methods. In our experiment, we use different normalization methods to make a comparison in next part.

### An adaptive term proximity normalization model

In the HRoc model, the Mean Average Precision (MAP) of the retrieval results is closely related to the *D* value. The traditional HAL model uses a fixed window size, so we need to make a large number of experiments to find the optimal *D* value and to improve the value of the MAP. While, this process wastes a lot of time and resources. In order to solve this problem, we need to make the model automatically find the most appropriate *D* value. In fact, a large number of experimental results show that the optimal *D* value is affected by the length of the document. For this reason, we try to use three different functions to fit the relationship between the *D* value and the length of the document, as is depicted in Table 2. The specific experimental results and analysis are given in next chapter.

**Table 2:** The three adaptive function

|*D*|		
*f*_1_(*dl*)	*f*_2_(*dl*)	*f*_3_(*dl*)
*dl*	*dl*∗(1+*dl*/*avg*(*dl*))	*dl*+*avg*(*dl*)/1+log2(*dl*/*avg*(*dl*))

### Test collections and evaluation metrics

In order to validate the effectiveness of our proposed model, we conduct a series of experiments on the standard TREC Clinical Decision Support Track collections. The document collection was updated to a more recent snapshot of PubMed Central (PMC)^2^ from 730*k* to 1.25 million full-text clinical medicine articles. The PMC collection contains articles published by PubMed Central in the year of 2016, including pmc-00, pmc-01, pmc-02 and pmc-03. We use pmc-00 and pmc-01 as test collections, pmc-02 and pmc-03 as experimental collections respectively. The topic numbers associated with each collection are presented in Table 3, and “No. of Doc” denotes the number of documents, “No. of Queries” means topic numbers.

**Table 3:** Collections statistics

Collection		No. of Doc	Size	No. of Queries
PMC	pmc-00	263175	16.9GB	30
	pmc-01	240347	15.8GB	30
	pmc-02	389431	21.2GB	30
	pmc-03	357047	19.6GB	30

**Fig. 3:**
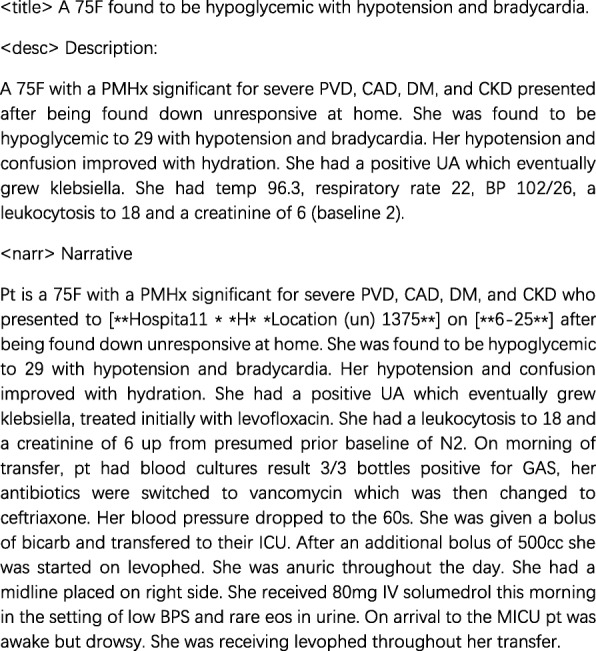
The example of topic style

For the collections, each topic contains three fields (title, description and narrative), and the example is introduced as follows in Fig. 3.

In the process of indexing and querying, queries without judgments are removed. For all test sets used, each term is stemmed by using Porters English stemmer. We leverage the Mean Average Precision (MAP) for the top 1000 documents to measure model performance in our experiments[36]. We take this metric as the primary evaluation metric in our experiments, which is typically used as the main official metric in the corresponding TREC evaluations. In addition, P@k can evaluation the relevance degree of the top-ranking documents, and we set *k*∈{5,10,20}. F1-Value can be represented as 2∗*PR*/(*P*+*R*), which is the harmonic average of the Recall (R) rate and the Precision (P) rate. Statistically significant evaluation metrics values based on the two-tailed paired *p*_*value* are computed at a 95*%* confidence level.

### Baseline models

Baseline models are very critical to verify the performance of the proposed methods. Firstly, we compare the proposed methods with the basic retrieval model BM25 and the KL-divergence language modeling (LM) retrieval [7,28]. Okapi BM25 is one of the most classical text retrieval algorithms. It is a probability weighted model. In BM25, the number of query term appear in documents (also called frequency) and the number of documents containing the query term are defined to assign weights. The corresponding weighting functions are as follows:

7 $$ {}\begin{aligned} {w(q_{i},D)}=\frac{(k_{1}+1)*tf(q_{i},D)}{K+tf(q_{i},D)}*\frac{(k_{3}+1)*qtf}{k_{3}*qtf}*log\left(\frac{N-df+0.5}{df+0.5}\right) \end{aligned}  $$

where *w*(*q*_*i*_,*D*) is the weight of query term qi in a document *D*, and *N* is the number of indexed documents in the collection. *k*1 and *k*3 are tuning constants that depend on the dataset used and possibly on the nature of the queries. *K* is equal to *k*1∗((1−*b*)+*b*∗*dl*/*avdl*), where *dl* is the length of the document, and *avdl* is the average document length. *df* is the number of documents containing a specific term. *tf*(*q*_*i*_,*D*) is the within-document term frequency, and *qtf* is the within-query term frequency.

Language model method is another classical algorithm in traditional information retrieval. Its basic idea is to estimate a language model for each document, and then sort the documents according to the possibility of query in the estimated language model. In particular, for the basic language model, we use a Dirichlet prior (with a hyper-parameter of *μ*^′^) to smooth the document language model as shown in Eq. (8) which generally results in better performance.

8 $$ {p(w|D)}=\frac{c(w_{D})+\mu'*p(w|c)}{\left|D\right|+ \mu'}  $$

where *c*(*w*_*D*_) is the frequency of query term w in document *D*, *p*(*w*|*c*) is the occurrence probability of term *w* in collection *C*, and |*D*| is the length of document *D*.

The primary estimation of relevance model is often called RM1 [7,41]. Essentially, RM1 uses the query likelihood *P*(*Q*|*D*) as the weight for document *D* and takes an average of the probability of each word given by each document language model. The relevance model *P*(*w*|*Q*) is commonly used to estimate the feedback language model *θ*_*F*_. And in order to improve performance [7,41], *θ*_*F*_ is generally interpolated into the original query model *θ*_*Q*_. However, it only captures the candidate terms’ distribution, but neglects the co-occurrence distribution between query terms and expansion terms. We make a comprehensive comparison with BM25+Rocchio and DLM+RM3, and a detailed analysis in our preliminary experiments.

For state-of-the-art PRF models, we compare the methods of PRoc2, PRoc3 and TF-PRF with the proposed method. The method of PRoc2 uses Gaussian kernel that shown to be effective in most cases, while PRoc3 model is similar to our method. Concerning the fact that PRoc2 and PRoc3 are more effective than PRoc1 [29], we would employ the former two methods to compare with the proposed methods in the experiments. Additionally, TF-PRF [30] is proposed by incorporating three different term frequency transformation methods. These two methods are representatives of the state-of-the-art models, which are capable of achieving the best IR performance on most of the standard TREC datasets.

### Parameter settings

In our model, several controlling parameters should be tuned for optimal results. Fairly, to find the optimal parameter settings, the following parameter settings for both baselines and the proposed model are used. And the related settings is well-known in IR for establishing strong baselines. First, in BM25, the value of *b* is swept from 0 to 1.0 with an interval of 0.1, and *k*_1_ and *k*_3_ are set to 1.2 and 8 respectively. Second, the Dirichlet prior smoothing *μ* (*μ*∈{500,600,…,2000}) in language model are then used to retrieve the documents. In other traditional medicine IR research, medicine data sets generally do not allow many candidate expansion terms based on practical application experience. So we sweep the number of feedback documents *N* and the feedback term |*T*_*f*_| from {10,20,…,50}. Finally, the HAL parameter *D* is set as *D*∈{0,100,…,2500}, and the interpolation parameter *α*,*β* are set as *α*,*β*∈{0.0,0.1,…,1.0}. In addition, we use 2-fold cross-validation to evaluate the proposed approaches, in which the TREC queries on each collection are partitioned into two sets by the parity of their numbers, and then the parameters learned from the training data set are applied to the test data set for evaluation purpose.

## Results and discussion

### Comparison with pRF basic models

After comparing the proposed methods with essential retrieval model BM25 and KL-divergence language modeling (LM) retrieval, we show the experimental results in Table 4.

**Table 4:** Performance of basic retrieval models in certain metrics

Basic models	MAP	P@5	P@10	P@20	F1
BM25	0.0448	0.2533	0.2467	0.2100	0.0739
DLM	0.0424	0.2800	0.2367	0.2100	0.0786

As is shown in Table 4, BM25 performs slightly better with Dirichlet prior in terms of MAP and P@10 metrics, and LM is superior to it in the rest of metrics. These two basic models perform comparatively, without observing significant different.

Next, “BM25+Rocchio”, the combination of BM25 and Rocchio’s model, and relevance language model (RM3) are used as two strong baselines in this paper. These two methods can achieve better retrieval performance in most cases. Using them as the basic models of the PRF baselines is reasonable. Thus, we use them to compare the proposed model.

We demonstrate the results of baseline PRF models and the three proposed models (HRoc1, HRoc2, and HRoc3) with different evaluation metrics in Table 5. In particular, “*” and “+” indicate a statistically significant improvement over BM25+Rocchio and RM3 respectively (Wilcoxon signed-rank test with *p*<0.05). The bold style in row corresponding to the best result. And Rocchio is used with BM25 and RM3 is used with LM for fair comparison.

**Table 5:** Comparison with baseline PRF retrieval models in certain metrics

	BM25+Rocchio	RM3	HRoc1	HRoc2	HRoc3
MAP	0.0490	0.0540	**0.0651** ^∗+^ (32.8%,20.5%)	0.0647 ^∗+^ (32.0%,19.8%)	0.0642 ^∗+^ (31.0%,18.9%)
P@5	0.2733	0.2600	**0.2933** ^∗+^ (7.32%,12.8%)	0.2733 ^+^ (0.00%,5.11%)	0.2733 ^+^ (0.00%,5.11%)
P@10	0.2533	0.2467	**0.2733** ^∗+^ (7.89%,10.8%)	0.2667 ^∗+^ (5.29%,8.11%)	0.2567 ^∗+^ (1.34%,4.25%)
P@20	0.2167	0.2233	**0.2350** ^∗+^ (8.44%,5.24%)	0.2317 ^∗+^ (6.92%, 3.76%)	0.2317 ^∗+^ (6.92%, 3.76%)
F1	0.0853	0.0932	**0.1112** ^∗+^ (30.3%,19.3%)	0.1108 ^∗+^ (29.9%,18.9%)	0.1096 ^∗+^ (28.5%, 17.6%)

The values in parentheses represent the improvements over BM25+Rocchio and RM3 respectively. The best result obtained is shown in bold, and the superscripts “*” and “+” denote statistically significant improvements over BM25+Rocchio and RM3, respectively (Wilcoxon signed-rank test with *p*<0.05)

Table 5 shows that the average performance of proposed models is better than that of baseline models. First, it has been proved that both Rocchio and RM3 are effective. They are considered to be strong baselines in previous studies. Rocchio model is superior to the RM3 model in terms of P@5 and P@10 metrics, but RM3 model outperforms Rocchio model in terms of MAP, P@20 and F1-value. Second, among the three proposed models, HRoc1 performs better retrieval results than HRoc2 or HRoc3 in terms of all metrics. This outcome proves the general effectiveness of our model. Third, HRoc1 gets the best performance of all the other four models. HRoc1 achieves average improvements of 32.8*%*, 7.32*%*, 7.89*%*, 8.44*%* and 30.3*%* over BM25+Rocchio in terms of the five metrics in TREC medicine collection respectively. The proposed HRoc1 model achieves average improvements of 20.5*%*, 12.8*%*, 10.8*%*, 5.24*%* and 19.3*%* over RM3 in terms of the five metrics in TREC medicine collection respectively.

### Comparison with the recent Progress

Because HRoc1 achieves better retrieval performance than HRoc2 and HRoc3, we just compare HRoc1 model with the state-of-the-art PRF models and proximity based PRF model (PRoc2, PRoc3, TF-PRF) using different evaluation metrics. Table 6 records the corresponding experimental results. In particular, “*”, “+”, and “#” represents statistically significant improvement over PRoc2, PRoc3, and TF-PRF, respectively. The bold style in row corresponding to the best result.

**Table 6:** Comparison with the state-of-the-art PRF retrieval models in certain metrics

	PRoc2	PRoc3	TF-PRF	HRoc1
MAP	0.0600	0.0593	0.0580	**0.0651** ^∗+*#*^ (8.50%,9.78%,12.24%)
P@5	0.2867	0.2667	0.2600	**0.2933** ^∗+*#*^ (2.30%,9.97%,12.81%)
P@10	0.2533	0.2300	0.2467	**0.2733** ^∗+*#*^ (7.90%,18.83%,10.78%)
P@20	0.2417	0.2167	0.2317	0.2350 ^+*#*^ (-2.77%,8.44%,1.42%)
F1	0.1031	0.1020	0.1012	**0.1112** ^∗+*#*^ (7.86%,9.02%,9.88%)

The best result obtained is shown in bold, and the superscripts “*”, “+” and “#” denote statistically significant improvements over PRoc2, PRoc3 and TF-PRF, respectively (Wilcoxon signed-rank test with *p*<0.05). The values in parentheses are the improvements over RM3, BM25+Rocchio, PRoc2 and TF-PRF, respectively

We can clearly see from Table 6 that the average performance of HRoc1 model is significantly better than other models on whole collections in most cases. PRoc2, Proc3 and TF-PRF have proven effective in previous studies [29,30]. First, the PRoc2 model outperforms the PRoc3 and TF-PRF model on the MAP, P@5, P@10, P@20 and F1 metrics. Second, the HRoc1 model outperforms PRoc3 and TF-PRF in terms of all metrics. Then PRoc2 gets best result in P@20 metrics among all eight methods. Third, the proposed HRoc1 model achieves average improvements of 8.5*%*, 2.30*%*, 7.90*%* and 30.3*%* over PRoc2 on the MAP, P@5, P@10 and F1 metrics in TREC medicine collection respectively.

HRoc1 is obviously superior to PRoc2, PRoc3, and TF-PRF. In terms of P@10, it performs up to 7.9% improvement, which is significantly better than that on MAP. The results demonstrate that our model performs well, especially in applications that emphasize on top results. To conclude, our model is at least comparable to the latest progress in probabilistic model and language model framework in MAP, and can perform significantly better in P@5, P@10, P@20 and F1-value.

### Comparison with different d value

**Fig. 4:**
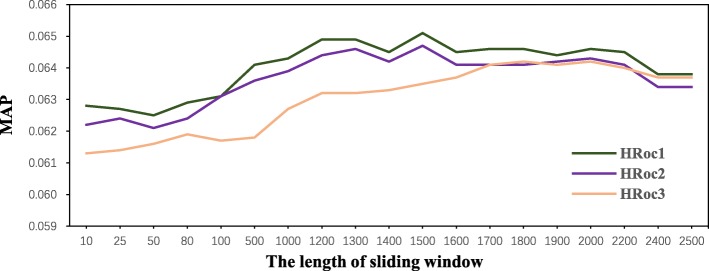
The performance of HRoc with different D values in MAP

In our preliminary experiments when smoothing methods, we introduced *D* value as a fixed value to measure proximity co-occurrence relationship of original query term and candidate query term. However, *D* value is also an important parameter in the HRoc model. We segment a document into a list of sliding windows, where each window has a fixed size. In our experiments, set *D* value varies from 10 to 2500. We use HRoc1, HRoc2, and HRoc3 to obtain the best result. Based on the results in MAP metrics, we get a Precision trend value, which is record in Fig. 4.

As is shown in Fig. 4, both HRoc1 and HRoc2 get their optimal Precision value where *D*=1500. We can observe a steady and slow decline trend through the two sides of the optimal value. Therefore, the methods of HRoc1 and HRoc2 are relatively stable models. HRoc3 do not obey this trend, so we consider that the *norm*_3_(*t*) normalization method is not suitable for processing the proximity weight result data of the medical sets.

### Comparison with adaptive d value

**Fig. 5:**
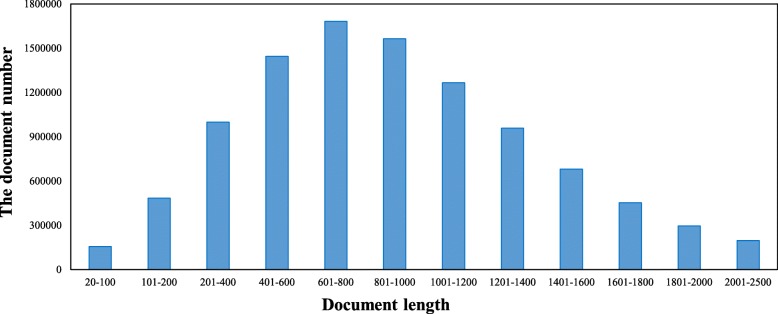
The statistical distribution of document lengths

The value of parameter *D* is fixed in the HAL model proposed previously, and the HAL weights of each document in the dataset are calculated according to the fixed value. In fact, the lengths of documents in the dataset are different from each other. In order to better research the relationship between *D* value and the length of the document, we calculate the lengths of the 125 million documents in the dataset, finding that the average length of all the documents is 816.3 and 87.7*%* of the document lengths are unevenly distributed ranging from 20 to 2500. Details can be found in Fig. 5.

**Fig. 6:**
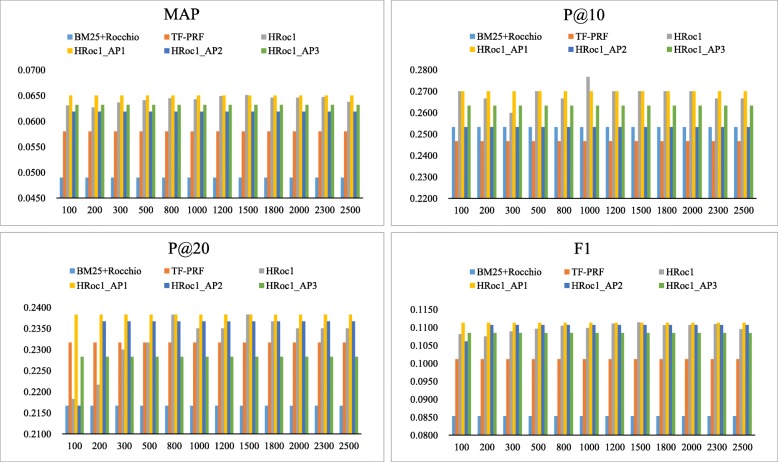
Performance comparison among 6 models in MAP, P@10, P@20 and F1 metrics

Under this kind of situation, using the same value of the parameter D to calculate the HAL weights for documents with different lengths is unreasonable. Thus, we assume that it might be more appropriate to set different D values for each document. To validate this assumption, we use three different automatic parameters to replace fixed D value. Due to HRoc1 achieves better retrieval performance, the following are three different adaptive functions of HRoc1 named HRoc1_AP1, HRoc1_AP2, and HRoc1_AP3 to make a comparison with the BM25 + Rocchio, TF- PRF and HRoc1 model, and D value of HRoc1 in the range of 100 to 2500. We get the MAP, P@10, P@20 and F1 trend values in Fig. 6 as following.

It is very intuitive to find that the first adaptive function model (HRoc1_AP1) is better than the original HRoc1 model under the evaluation metrics of MAP, P@20 and F1. HRoc1_AP1 is superior to other three models in terms of P@10 at most cases. The parameter D of the adaptive function in HRoc1_AP1 is the length of the document itself, which directly confirms our previous idea that setting different D value for each document. From the comparison results of the experiment, the most suitable setting for D value is document length (dl).

Since the required parameter (dl) is an existing constant when the adaptive function (HRoc1_AP1) is calculated, the algorithm complexity of adaptive function model is not increased comparing to the original model.

### Discussion

In this paper, we use five different comparing methods. In two basic retrieval models BM25+Rocchio and the KL-divergence language modeling (LM)+RM3, we get optimal values when we set *b*=0.5 and *μ*=2000.

We make experiments of PRoc2 in *σ*∈{10,30,50,100,500,1000,1500}. As a state-of-the-art model in proximity PRF method, PRoc2 gets optimal values when *σ*=50. In addition, HRoc1 achieves best result in all five different comparing methods. We get optimal value when *D*=1500. The method of *norm*_1_(*t*) is the most effective in proposed three normalization methods. Both sides of the optimal value demonstrate a steady and slow decline trend.

The effectiveness of the proposed methods can be proved by the experiments on TREC medicine collections. The modification to the PRF model leads to significant and substantial (up to 32.8*%*) improvements. Furthermore, the proposed proximity-based function outperforms the three well-known constraints (PRoc2, PRoc3, TF-PRF). It may due to the positional semantic information in clinical records and articles. For example, the name of the drugs can represent a disease, while an article with no appearance of the disease name can be judged to be relevant when the name of a drug is close to the location of the original query. However, this phenomenon also appears in other datasets, but with higher frequency in clinical medical collection. It may also due to the significant degree of candidate term in feedback documents, which are not only decided by term frequency because some clinical jargon only appear once.

### Limitations and future work

In this paper, a large number of research and experimental analysis on medical datasets, which makes the scope of application is not broad enough, the model method also has more in-depth optimization space.

Next, we will explore some other directions in the future work. First, we will do more experiments on other category collections, and therefore study more suitable relationships between *D* value and the length of document. Second, it is interesting to study on integrating the term’s co-occurrence into other models, especially deep learning models [42,43]. We also plan to evaluate our proposed methods on some real-world collections and applications.

## Conclusions

In this paper, we proposed an enhanced HAL-based Rocchio’s method. We integrate term’s proximity co-occurrence weight information into classic Rocchio’s model to improve retrieval performance. We then integrate three normalization methods into proposed HRoc1, HRoc2, and HRoc3 model. Proposed new method can measure the proximity co-occurrence relationships between a candidate term and a query term. Experimental results show that our model significantly outperforms the strong baseline PRF models in terms of MAP, P@10, P@20, and F1-value. Meanwhile, our proposed methods are comparable to the state-of-art model PRoc2, PRoc3, and TF-PRF.

Additionally, we carefully analyze the *D* value of our proposed three HRoc methods and get a tendency figure in MAP. When *D* value is equal to two times of average document length, it would get the highest point of the curve. The average length of the CDS track dataset is 816.3, and we naturally associate that the *D* value should be related to the length of the document. Then, we make statistics and analysis on the document length of the whole dataset, and propose three adaptive functions related to *dl* to replace the *D* value and make three non-parametric adjacent normalization models. The experimental results show that the first non-parametric adjacent normalization model is not only comparable to the previous model, but also does not increase the complexity of the algorithm. At the same time, the adaptive function model has less hyper parameters than the original model, which improves the universality of the model.

## Data Availability

The presented results are available.

## Abbreviations

BM25: Best match25
CDS: Clinical decision support
DFR: Divergence from randomness models
EHR: Electronic health record
HAL: Hyperspace analogue to language
IR: Information retrieval
MAP: Mean average precision
PMC: PubMed central
PRF: Pseudo relevance feedback
PRoc: Proximity-based feedback framework
TREC: Text retrieval conference

## Footnotes

1 http://www.trec-cds.org/

2 https://www.ncbi.nlm.nih.gov/pmc/tools/openftlist/
